# A Treatment Planning Study of Stereotactic Body Radiotherapy for Atrial Fibrillation

**DOI:** 10.7759/cureus.678

**Published:** 2016-07-11

**Authors:** Ping Xia, Rupesh Kotecha, Naveen Sharma, Martin Andrews, Kevin L Stephans, Carlos Oberti, Sara Lin, Oussama Wazni, Patrick Tchou, Walid I Saliba, John Suh

**Affiliations:** 1 Department of Radiation Oncology, Cleveland Clinic; 2 Department of Cardiovascular Medicine, Cleveland Clinic; 3 Department of Cardiovascular Medicine, Cleveland; 4 Cleveland Clinic

**Keywords:** stereotactic body radiotherapy, atrial fibrillation, treatment planning, hypofractionation, radiotherapy

## Abstract

Purpose: To explore the feasibility of using stereotactic body radiotherapy (SBRT) to irradiate the antra of the four pulmonary veins while protecting nearby critical organs, such as the esophagus.

Materials and Methods: Twenty patients who underwent radiofrequency catheter ablation for atrial fibrillation were selected. For each patient, the antra of the four pulmonary veins were identified as the target volumes on a pre-catheterization contrast or non-contrast CT scan. On each CT scan, the esophagus, trachea, heart, and total lung were delineated and the esophagus was identified as the critical organ. For each patient, three treatment plans were designed with 0, 2, and 5 mm planning margins around the targets while avoiding overlap with a planning organ at risk volume (PRV) generated by a 2 mm expansion of the esophagus. Using three non-coplanar volumetric modulated arcs (VMAT), 60 plans were created to deliver a prescription dose of 50 Gy in five fractions, following the SBRT dose regimen for central lung tumors. With greater than 97% of the planning target volumes (PTV) receiving the prescription doses, we examined dosimetry to 0.03 cc and 5 cc of the esophagus PRV volume as well as other contoured structures.

Results: The average PTV-0 mm, PTV-2 mm, and PTV-5 mm volumes were 3.05 ± 1.90 cc, 14.70 ± 5.00 cc, and 40.85 ± 10.20 cc, respectively. With three non-coplanar VMAT arcs, the average conformality indices (ratio of prescription isodose volume to the PTV volume) for the PTV-0 mm, PTV-2 mm and PTV-5 mm were 4.81 ± 2.0, 1.71 ± 0.19, and 1.23 ± 0.08, respectively. Assuming patients were treated under breath-hold with 2 mm planning margins to account for cardiac motion, all plans met esophageal PRV maximum dose limits < 50 Gy to 0.03 cc and 16 plans (80%) met < 27.5 Gy to 5 cc of the esophageal PRVs. For PTV-5 mm plans, 18 plans met the maximum dose limit < 50 Gy to 0.03 cc and only two plans met the maximum dose limit < 27.5 Gy to 5 cc of the esophageal PRV.

Conclusions: The anatomical relationship between the antra of the four pulmonary veins and the esophagus varies from patient to patient. Adding 2 mm planning margins and a 2 mm PRV to the esophagus can meet the dose constraints developed for SBRT central lung tumors. Future studies are needed to validate the safety and efficacy of the planning dose, tolerance dose to normal cardiac tissue, and adequate planning margins.

## Introduction

Atrial fibrillation is the most common arrhythmia encountered in medical practice and is a growing global health concern with a 19% increase over the last 20 years and 5 million new cases diagnosed each year worldwide [1-3]. In the United States, it is estimated that 2.3 million adults are diagnosed with atrial fibrillation. This number is projected to increase to 5.6 million by 2050 with more than 50% of patients being 80 years or older [4]. For patients who are treated for rhythm control, treatment options include medical therapies aimed at suppressing the arrhythmia or an ablative procedure to destroy the arrhythmogenic source itself. For atrial fibrillation cases that are refractory to medical therapy, catheter ablation (CA) through either radiofrequency or cryothermy is an important treatment option. The aim of the procedure is to eliminate the arrhythmogenic tissue by either heating (radiofrequency) or cooling (cryothermy) [2]. Similarly, using X-rays, stereotactic radiosurgery (SRS) can eliminate arrhythmogenic tissue. Stereotactic radiosurgery has a long history of successful treatment for non-cancer conditions, such as trigeminal neuralgia and arteriovenous malformations [5].

Major complications and even death with CA procedures are risks [2, 6-8]. The procedures are experience-dependent, invasive, and complex. For predominantly younger and healthier patients, the reported major complications occurring during the ablation procedures are approximately 5% [2, 6-8]. However, for elderly patients with other medical comorbidities, the risks of the procedures may be even higher [2, 6-8].

Using focused radiation for patients with medically inoperable early-stage lung cancer, stereotactic body radiosurgery (SBRT) has achieved excellent local control [9-10]. It is postulated that for patients with refractory atrial fibrillation and other medical comorbidities, for whom the CA procedure may pose a high risk, SRS may be an alternative option of treatment [11]. The SRS procedure is a non-invasive, outpatient procedure. With a CyberKnife platform, the feasibility of SRS was tested on scar tissue creation in the cavotricuspid isthmus and pulmonary vein atria in normal swine models [11]. Using a single patient image data set, Ipsen, et al. [12] conducted a single fraction planning study with variable planning margins to compensate for breathing motion observed on real-time MRI. Using patient CT images acquired for the CA procedures, we conducted a feasibility treatment planning analysis with multiple fractionations similar to stereotactic body radiation to explore whether the esophagus dose constraints for SBRT lung treatment can be achieved.

The treatment volumes defined for this planning study mimic volumes of CA, which are in anatomical proximity to the esophagus. The objective of this planning study is to investigate what planning margins can safely accommodate the esophagus tolerance dose using clinically derived SBRT dose constraints to the esophagus for centrally located early-stage lung cancer [13-15] while accounting for cardiac and respiratory motion. 

The study was approved by the Cleveland Clinic Institutional Review Board, protocol # 15-005. A waiver of informed patient consent was approved for the study.

## Materials and methods

### Delineation of target volumes and critical organs

Twenty patients who underwent radiofrequency CA for atrial fibrillation were randomly selected. For each patient, the antra of the four pulmonary veins were identified as the target volume on the pre-catheterization contrast or non-contrast CT scan. On each CT scan, the esophagus, trachea, heart, and total lung were delineated with the esophagus identified as the critical organ. The esophagus was uniformly expanded by 2 mm to create the planning organ-at-risk volume (PRV) in order to account for residual respiratory and cardiac motion-induced esophageal motion. The physical density of the contrast agents administered during acquisition of the planning CT was overridden to 1.0 g/cm^3 ^because no contrast agent would be administered during treatment. 

### Radiation dose

For patients with early-stage, non-small cell, centrally located lung cancers, the esophagus volume dose constraints from the Radiation Therapy Oncology Group (RTOG) 0813 [14] are a maximum point dose of 52.5 Gy and a 5 cc dose limit of 27.5 Gy. Our previous clinical experience found no significant late esophageal toxicity when the esophageal point dose (to 0.03 cc of the volume) was less than 50 Gy and the dose to 1 cc of the esophagus volume was less than 45 Gy [15]. Because of heart motion and the potential displacement of the esophagus, we applied these dose constraints to the esophagus PRV instead of the esophagus itself. These dose limits were based on the five fraction scheme with a prescription of 50 Gy. Previous pre-clinical studies [11, 16] showed that a single fraction of 32.5 Gy was necessary to achieve transmural scarring of the myocardium, similar to CA. Directly applying a linear-quadratic (LQ) model with alpha/beta ratios of 3 Gy (late effect) and 10 Gy, 32.5 Gy in a single fraction is equivalent to 230.75 Gy and 115.1 Gy in 2 Gy per fraction, respectively. It is, however, known that the LQ model may over-estimate the total dose in fraction size > 8-10 Gy [17]. Alternatively, radiobiological modeling studies have determined that a biological effective dose (BED) of 34 Gy in a single fraction is equivalent to 48 Gy in four fractions [18-19]. We, therefore, chose a prescription dose of 50 Gy in five fractions, for which we have esophageal clinical tolerance dose experience.

### Planning technique

For each patient, three treatment plans were designed with 0, 2, and 5 mm planning margins to the target volumes (referred to as PTV-0 mm, PTV-2 mm, and PTV-5 mm, respectively) while avoiding overlaps of the esophagus PRVs. Three non-coplanar volumetric modulated arcs (VMAT) were utilized for each plan with a full arc at the couch angle of 0°, a partial arc (from 40° to 182°) at the couch angle of 20°, and a partial arc (from 320° to 178°) at the couch angle of 340°. A total of 60 plans were created utilizing the automatic planning module in the Pinnacle treatment planning system (Philips Radiation Oncology Systems, Fitchburg, WI). To create a highly conformal plan with the auto-planning module, we expanded each PTV by an additional 2 mm and 2 cm, named as PTVs_2 mm and PTVs_2 cm, respectively. From the PTVs_2 cm, we created a ring structure of 2 cm, named 2 cm_ring. For all PTVs, the planning dose objectives used for the auto-planning module are listed in Table 1. These planning objectives were applied to all 60 plans. Because of small PTVs, the final dose calculation resolution was 2 mm using the collapsed cone convolution algorithm. A high-definition multileaf collimator was used for beam shaping with a minimum leaf width of 0.25 cm. 

**Table 1:** An Example of Planning Dose Objective Input into the Auto-Planning for PTV-2 mm Plans ROI = region of interest; DVH = dose volume histogram; PTV = planning target volume, PRV = planning organ at risk volume

ROI	Type	Dose (Gy)	Vol (%)	Priority	Compromise
PTV-2 mm	Target	50			
2 mm_Ring	Mean Dose	45		High	Allow
5 mm_Ring	Max Dose	40		High	Allow
2 cm_Ring	Max Dose	22		High	Allow
Esophagus_2 mm PRV	Max Dose	40		High	Allow
Esophagus_2 mm PRV	Max DVH	35	5%	High	Allow
Esophagus	Max Dose	48		High	Allow
Spinal Cord	Max Dose	15		High	Allow
Spinal Cord	Max DVH	13	3%	High	Allow

### Plan evaluations

With greater than 97% of the planning target volumes receiving the prescription dose of 50 Gy, the planning constraints for the esophagus PRV were set as: maximum dose to 0.03 cc < 50 Gy, 1 cc < 45 Gy, 2 cc < 33.5 Gy, and 5 cc of the esophagus PRV < 27.5 Gy. It should be noted these dose constraints from RTOG 0813 [14] and other studies [13, 15, 20-21] were applied to the esophagus, not the esophagus PRV. Because of cardiac motion, we applied these dose constraints conservatively. Multiply defined endpoint doses to other sensitive structures, such as the heart, aorta, left atrium, trachea, lung, and spinal cord, were reported as well.

## Results

**Figure 1:**
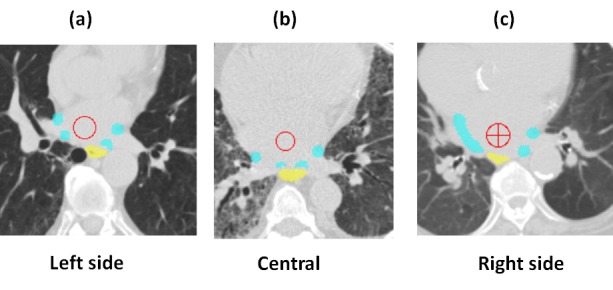
The esophagus had overlapped or was within a short distance to the target on the (a) left side, (b) centrally, and (c) right side.

**Figure 2:**
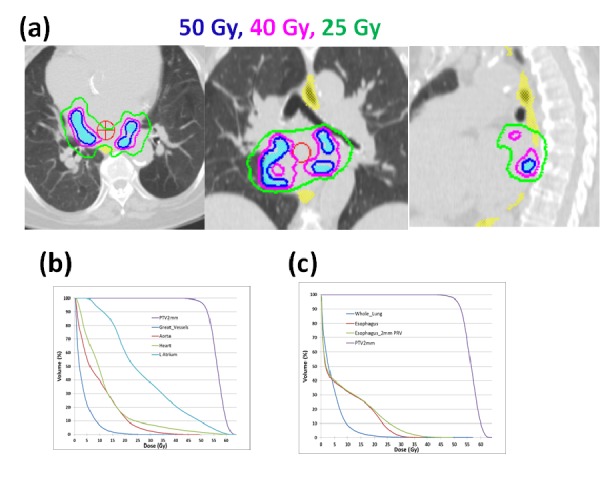
A) Three-dimensional dose distributions for a selected patient and B-C) dose volume histograms.

In the human heart, the left atrium is immediately anterior to the esophagus and the pulmonary veins are in close proximity to the esophagus. The anatomical relationship between the target and the esophagus varies from patient to patient, as listed in Table 2. Figure 1A-1C shows three examples of anatomic variations between the target and the esophagus. In Figure 1A-1C, the esophagus had overlapped or was within a short distance to the target on the (a) left side, (b) centrally, and (c) right side. Among 20 patients, 15 patients (75%) had target volumes that overlapped with the esophagus 2 mm PRV, and five patients (25%) had a minimum distance that varied from 1 mm to 5.7 mm. The majority of patients (13 out 20, 65%) had an overlap or the shortest distance occurring on the left side, four patients on the right side, and three patients centrally. Figure 2A-2C shows examples of dose distributions in three dimensions and dose-volume histograms for a selected patient dataset. Among 20 patient datasets, the average PTV-0 mm, PTV-2 mm, and PTV-5 mm volumes were 3.05 ± 1.90 cc, 14.70 ± 5.00 cc, and 40.85 ± 10.20 cc (mean ± standard deviation), respectively. With three non-coplanar VMAT arcs, the average conformality indices (ratio of prescription isodose volume to the PTV volume) for PTV-0 mm, PTV-2 mm, and PTV-5 mm were 4.81 ± 2.0, 1.71 ± 0.19, and 1.23 ± 0.08, respectively.

**Table 2:** Anatomical Relationship Variations Between the Esophagus and the Target Volume

Patient ID	Distance (mm)	Side
1	Overlap	Central
2	Overlap	L
3	5.7	L
4	Overlap	L
5	Overlap	L
6	Overlap	L
7	Overlap	R
8	1.8	L
9	2.6	L
10	Overlap	L
11	2.7	L
12	Overlap	L
13	Overlap	L
14	1.0	R
15	Overlap	Central
16	Overlap	L
17	Overlap	Central
18	Overlap	L
19	Overlap	R
20	Overlap	R

**Figure 3:**
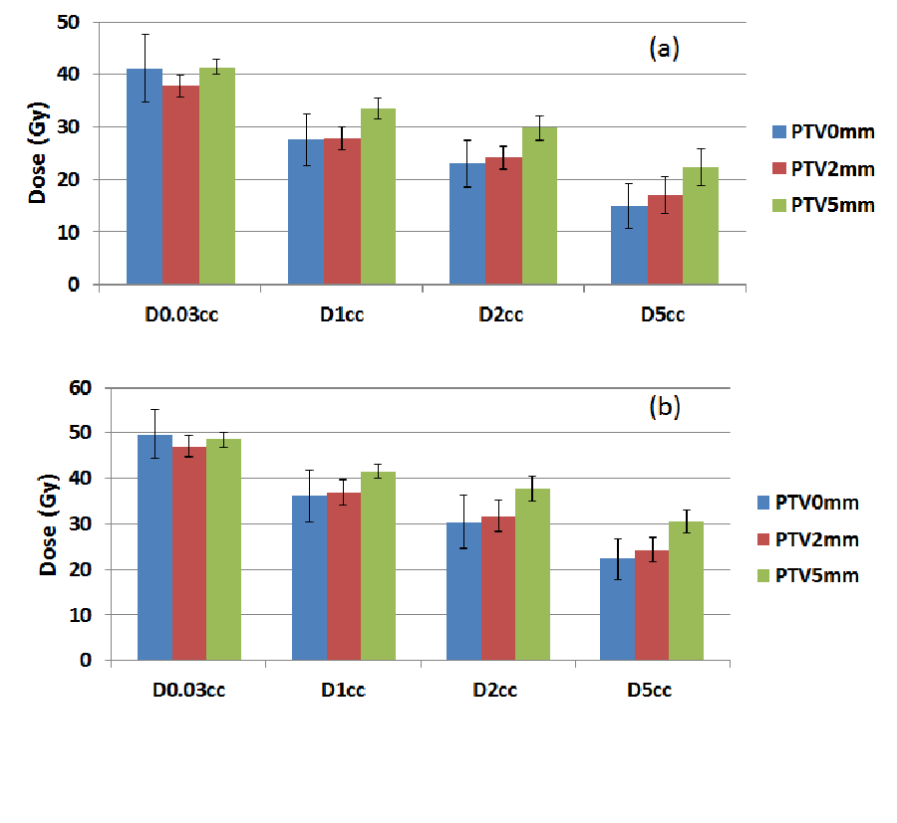
A) The average doses of D0.03 cc, D1 cc, D2 cc, and D5 cc of esophagus and B) esophagus PRV and their standard deviations for PTV-0 mm, PTV-2 mm, and PTV-5 mm plans.

Figure 3A-3B shows the average doses of D0.03 cc, D1 cc, D2 cc, D5 cc of the esophagus, and esophagus PRV and their standard deviations for PTV-0 mm, PTV-2 mm, and PTV-5 mm plans. Table 3 lists the number of plans that exceeded the dose limits of D0.03 cc < 50 Gy, D1 cc < 45 Gy, D2 cc < 33.5, and D5 cc < 27.5 Gy. Because all plans were normalized to have 97% of the PTVs receiving the prescription dose of 50 Gy, PTV-0 mm plans had the smallest PTV volumes, resulting in the highest D0.03 cc for the esophagus and esophagus 2 mm PRV when compared to those in PTV-2 mm and PTV-5 mm plans. If the patients were treated during breath-hold using 2 mm planning margins to account for the cardiac motion, all PTV-2 mm plans would meet the esophageal dose limits of D0.03 cc < 50 Gy, D1 cc < 45 Gy, D2 cc < 33.5 Gy, and D5 cc < 27.5 Gy. For the esophagus PRV, dose limits for D0.03 cc and D1 cc were met by all plans, but the dose limits for D2 cc and D5 cc were exceeded by eight (40%) plans and four plans (80%), respectively. 

**Table 3:** A Summary of the Number of Plans Exceeded Esophagus and Esophagus 2 mm PRV Dose Tolerance Among PTV-0 mm, PTV-2 mm, and PTV-5 mm Plans

		Esophagus				Esophagus 2 mm PRV			
	End Point	D0.03 cc	D1 cc	D2 cc	D5 cc	D0.03 cc	D1 cc	D2 cc	D5 cc
	Dose Limits	< 50 Gy	< 45 Gy	< 33.5 Gy	< 27.5 Gy	< 50 Gy	< 45 Gy	< 33.5 Gy	< 27.5 Gy
PTV-0 mm	# plans exceeded	0	0	0	0	14	0	7	1
PTV-2 mm	# plans exceeded	0	0	0	0	0	0	8	4
PTV-5 mm	# plans exceeded	0	0	1	2	2	0	19	18

**Figure 4:**
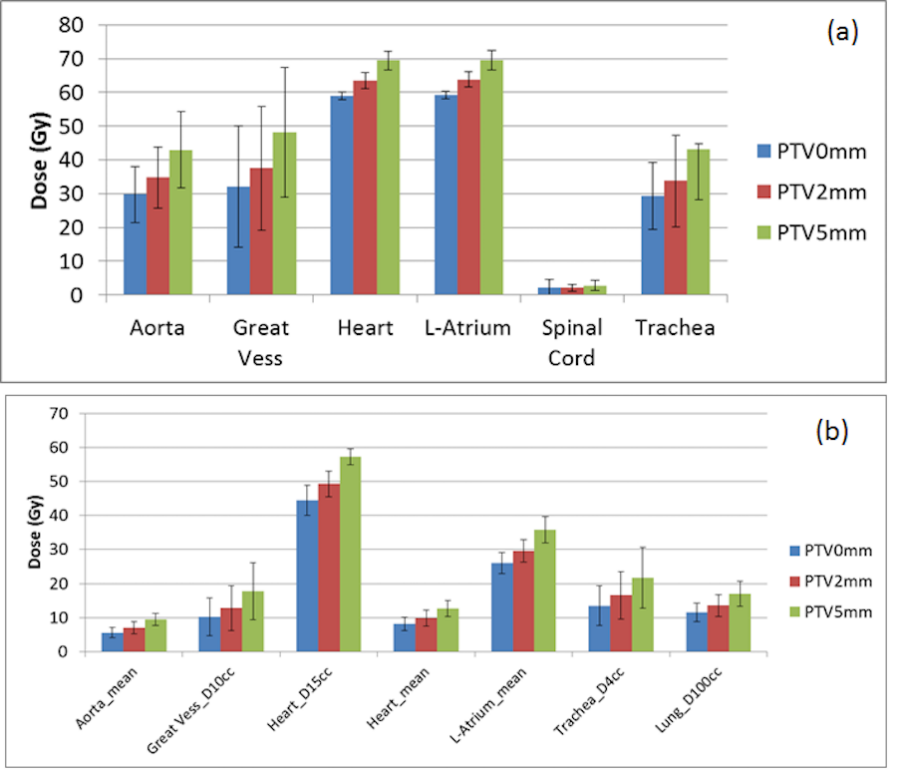
A) The average maximum doses and B) the average volume specified doses to listed organs and their standard deviations.

Figure 4A shows the average maximum doses (defined as D0.03 cc to all structures, except the spinal cord, which is defined as the dose to 0.35 cc) to the aorta, great vessels, heart, left atrium, spinal cord, and trachea and their standard deviations for PTV-0 mm, PTV-2 mm, and PTV-5 mm plans. Figure 4B shows the average specified volume doses to the aorta, great vessels, heart, left atrium, trachea, and total lung and their standard deviations for PTV-0 mm, PTV-2 mm, and PTV-5 mm plans. According to the report of the American Association of Physicists in Medicine (AAPM) Task Group 101, tolerance doses to other normal tissues were listed in Table 4 and the number of PTV-2 mm plans, which exceeded the listed tolerance, were also listed [19]. 

**Table 4:** A Summary of the Number of PTV-2 mm Plans Exceeded Tolerance Doses of AAPM TG 101 ROI - region of interest

ROI	Endpoint	# Plans
		Exceeded
Aorta	D0.03 cc	NA
Aorta	Dmean	NA
Great Vessels	D0.03 cc < 53 Gy	4
Great Vessels	D10 cc < 47 Gy	0
Heart	D0.03 cc < 38 Gy	20
Heart	D15 cc < 32 Gy	20
Heart	Dmean	NA
L Atrium	D0.03 cc	NA
L Atrium	Dmean	NA
Spinal Cord	D0.35 cc < 23 Gy	0
Trachea	D0.03 cc < 40 Gy	5
Trachea	D4 cc < 16.5 Gy	10
Whole Lung	D100 cc < 13.5 Gy	10

## Discussion

In this paper, we conducted a treatment planning study to explore a potential new application of stereotactic body radiation therapy for atrial fibrillation. The major technical challenges in irradiating cardiac-targeted tissue are the management of breathing and cardiac motions and unknown radiation toxicities to normal cardiac tissues, such as the aorta, great vessels, atria, and heart, after hypofractionated radiation. Among all organs at risk, we identified the esophagus as the most critical organ for the treatment of atrial fibrillation with focused radiation due to its proximity to the radiation target volume while documenting the radiation dose to other normal organs. Following our clinical SBRT experience for early stage centrally located lung tumors, we chose a prescription dose of 50 Gy in five fractions, for which we have clinical esophageal dose tolerance experience [15]. In this series, we found no significant late esophageal toxicity when the dose limits for the maximum dose (D0.03 cc) < 50 Gy and D1cc < 45 Gy were met. Other studies suggested the dose limits to D2 cc and D5 cc for the esophagus, although the correlation between late esophagus toxicity and dose limits to D2 cc (< 33.5 Gy) and D5 cc (< 27.5 Gy) of the esophagus were not conclusive [13-15, 20-21]. With the maximum dose limits (D0.03 cc < 50 Gy and D1cc < 45 Gy), we found that the PTV-2 mm plans may provide adequate protection to the esophagus while a 2 mm PTV margin may be a reasonable magnitude to account for the heart motion.

Concerning cardiac motion and radiation dose delivery precision, Gardner, et al. implanted thermoluminescent dosimeter (TLD) crystals onto the surface of canine hearts and transferred metal-oxide-semiconductor field-effect transistor (MOSFET) sensors via a catheter in canine and porcine models close to an ablation target volume to measure the difference between the delivered and planned radiation doses [23]. Planned with an internal left atrium target volume derived from cardiac-gated CT scans, they found the measured doses were within 10% of the planned doses using either of the dose-monitoring techniques. Additional research also demonstrates that radiosurgical targeting of cardiac motion is, in fact, more predictable than respiratory motion [24]. Real-time MRI scans of healthy individuals have revealed that left atrial motion is 7.8 mm in the superior, 1.6 mm in anterior, and 0.7 mm in the left direction. In our study, we did not have cardiac-gated CT images for these patients but simply applied uniform margins of 2 mm and 5 mm. Considering a quadratic relationship between the organ motion and treatment set-up uncertainties, the 2 mm planning margin can adequately account for 1.6 mm cardiac motion in anterior-posterior and lateral directions and 1 mm setup uncertainty. However, the real-time MRI study [24] was performed during a normal heartbeat, likely different from the irregular motion for patients with atrial fibrillation.

The rhythm of cardiac motion for patients with atrial fibrillation can be unpredictable, but the motion magnitude may be similar to healthy patients. In this study, we assumed that the respiratory motion could be perfectly controlled under active breathing motion control and assumed a perfect patient set-up for five daily treatments. These assumptions might be difficult to achieve clinically. On the other hand, if we further expanded the PTV margin to 5 mm, taking into account the cardiac motion, imperfect breathing motion control, and uncertainties in daily patient set-up, then two PTV-5 mm plans would exceed the maximum dose limit of D0.03 cc < 50 Gy and most PTV-5 mm plans would exceed the D2 cc and D5 cc dose limits to the 2 mm PRV esophagus. Because automated treatment planning was used for this study, it is also possible than an experienced human planner could further reduce the dose to the esophageal PRV while maintaining target coverage.

In a future study, non-uniform planning margins may be considered, especially to increase the planning margins in the superior and inferior directions. Since the shortest distance between the esophagus and the targets are in the anterior and posterior directions, we do not anticipate a drastic increase in the esophagus dose by increasing a superior-inferior planning margin in the targets. The doses to other normal structures may increase, which will require further investigation. Since the radiation target volumes in the present study modeled CA, a potential alternative would be redefining the target volume distance away from the esophagus while still achieving the treatment goal of transmural scarring of the myocardium. 

Directly applying an esophageal toxicity profile from cancer patients to non-cancer patients is subject to debate as we often do not know the late normal tissue toxicities for cancer patients. The best data we have for cardiac toxicity is from the report of the American Association of Physicists in Medicine (AAPM) Task Group 101 [22]. As the authors of the report pointed out, the suggested tolerance doses to these normal tissues are not fully validated and only serve as a first approximation. A recent study on the rectal toxicity from SBRT prostate treatment indicated that for a tubular structure, such as the rectum and esophagus, instead of a volume dose, the radiation dose to the circumference of the structure may be a better prediction of toxicity because of the ability of repair by the adjacent stem cells [25]. Our future study will also report on the esophagus circumference dose. Furthermore, the tolerance doses to other substructures of the heart, such as the aorta, left atrium, ventricles, and mitral valves, were not listed in the AAPM Task Group 101 report and, therefore, requires further investigation [22]. 

## Conclusions

The anatomical relationship between the antra of the four pulmonary veins and the esophagus varies from patient to patient. Adding 2 mm planning margins and a 2 mm PRV to the esophagus can meet the dose constraints developed for SBRT central lung tumors. Future studies are needed to explore different target volume strategies to validate the safety and efficacy of the planning dose to the target volumes, the tolerance doses to the normal cardiac tissue, and adequate planning margins. 

## References

[1] Chugh SS, Havmoeller R, Narayanan K, Singh D, Rienstra M, Benjamin EJ, Gillum RF, Kim YH, McAnulty JH Jr, Zheng ZJ, Forouzanfar MH, Naghavi M, Mensah GA, Ezzati M, Murray CJ (2014). Worldwide epidemiology of atrial fibrillation: A Global Burden of Disease 2010 study. Circulation.

[2] Maan A, Shaikh AY, Mansour M, Ruskin JN, Heist EK (2011). Complications from catheter ablation of atrial fibrillation: a systematic review. Crit Pathw Cardiol.

[3] Naccarelli GV, Varker H, Lin J, Schulman KL (2009). Increasing prevalence of atrial fibrillation and flutter in the United States. Am J Cardiol.

[4] Go AS, Hylek EM, Phillips KA, Chang Y, Henault LE, Selby JV, Singer DE (2001). Prevalence of diagnosed atrial fibrillation in adults: national implications for rhythm management and stroke prevention: the AnTicoagulation and Risk Factors in Atrial Fibrillation (ATRIA) Study. JAMA.

[5] Guo S, Chao ST, Reuther AM, Barnett GH, Suh JH (2008). Review of the treatment of trigeminal neuralgia with Gamma Knife radiosurgery. Stereotact Funct Neurosurg.

[6] Dagres N, Hindricks G, Kottkamp H, Sommer P, Gaspar T, Bode K, Arya A, Husser D, Rallidis LS, Kremastinos DT, Piorkowski C (2009). Complications of atrial fibrillation ablation in a high-volume center in 1,000 procedures: still cause for concern?. J Cardiovasc Electrophysiol.

[7] Marrouche NF, Dresing T, Cole C, Bash D, Saad E, Balaban K, Pavia SV, Schweikert R, Saliba W, Abdul-Karim A, Pisano E, Fanelli R, Tchou P, Natale A (2002). Circular mapping and ablation of the pulmonary vein for treatment of atrial fibrillation: impact of different catheter technologies. J Am Coll Cardiol.

[8] Spragg DD, Dalal D, Cheema A, Scherr D, Chilukuri K, Cheng A, Henrikson CA, Marine JE, Berger RD, Dong J, Calkins H (2008). Complications of catheter ablation for atrial fibrillation: incidence and predictors. J Cardiovasc Electrophysiol.

[9] Timmerman R, Paulus R, Galvin J, Michalski J, Straube W, Bradley J, Fakiris A, Bezjak A, Videtic G, Johnstone D, Fowler J, Gore E, Choy H (2010). Stereotactic body radiation therapy for inoperable early stage lung cancer. JAMA.

[10] Videtic GM, Stephans K, Reddy C, Gajdos S, Kolar M, Clouser E, Djemil T (2010). Intensity-modulated radiotherapy-based stereotactic body radiotherapy for medically inoperable early-stage lung cancer: excellent local control. Int J Radiat Oncol Biol Phys.

[11] Blanck O, Bode F, Gebhard M, Hunold P, Brandt S, Bruder R, Grossherr M, Vonthein R, Rades D, Dunst J (2014). Dose-escalation study for cardiac radiosurgery in a porcine model. Int J Radiat Oncol Biol Phys.

[12] Ipsen S, Blanck O, Oborn B, Bode F, Liney G, Hunold P, Rades D, Schweikard A, Keall PJ (2014). Radiotherapy beyond cancer: target localization in real-time MRI and treatment planning for cardiac radiosurgery. Med Phys.

[13] Chang JY, Li QQ, Xu QY, Allen PK, Rebueno N, Gomez DR, Balter P, Komaki R, Mehran R, Swisher SG, Roth JA (2014). Stereotactic ablative radiation therapy for centrally located early stage or isolated parenchymal recurrences of non-small cell lung cancer: how to fly in a "no fly zone". Int J Radiat Oncol Biol Phys.

[14] (2016). RTOG 0813 Protocol Information: Seamless Phase I/II Study of Stereotactic Lung Radiotherapy (SBRT) for Early Stage, Centrally Located, Non-Small Cell Lung Cancer (NSCLC) in Medically Inoperable Patients. http://www.rtog.org/ClinicalTrials/ProtocolTable/StudyDetails.aspx?study=0813.

[15] Stephans KL, Djemil T, Diaconu C, Reddy CA, Xia P, Woody NM, Greskovich J, Makkar V, Videtic GM (2014). Esophageal dose tolerance to hypofractionated stereotactic body radiation therapy: risk factors for late toxicity. Int J Radiat Oncol Biol Phys.

[16] Sharma A, Wong D, Weidlich G, Fogarty T, Jack A, Sumanaweera T, Maguire P (2010). Noninvasive stereotactic radiosurgery (CyberHeart) for creation of ablation lesions in the atrium. Heart Rhythm.

[17] Fowler JF (1989). The linear-quadratic formula and progress in fractionated radiotherapy. Br J Radiol.

[18] Park C, Papiez L, Zhang S, Story M, Timmerman RD (2008). Universal survival curve and single fraction equivalent dose: useful tools in understanding potency of ablative radiotherapy. Int J Radiat Oncol Biol Phys.

[19] Videtic GM, Stephans KL, Woody NM, Reddy CA, Zhuang T, Magnelli A, Djemil T (2014). 30 Gy or 34 Gy? Comparing 2 single-fraction SBRT dose schedules for stage I medically inoperable non-small cell lung cancer. Int J Radiat Oncol Biol.

[20] Abelson JA, Murphy JD, Loo BW Jr, Chang DT, Daly ME, Wiegner EA, Hancock S, Chang SD, Le QT, Soltys SG, Gibbs IC (2012). Esophageal tolerance to high-dose stereotactic ablative radiotherapy. Dis Esophagus.

[21] Cox BW, Jackson A, Hunt M, Bilsky M, Yamada Y (2012). Esophageal toxicity from high-dose, single-fraction paraspinal stereotactic radiosurgery. Int J Radiat Oncol Biol Phys.

[22] Benedict SH, Yenice KM, Followill D, Galvin JM, Hinson W, Kavanagh B, Keall P, Lovelock M, Meeks S, Papiez L, Purdie T, Sadagopan R, Schell MC, Salter B, Schlesinger DJ, Shiu AS, Solberg T, Song DY, Stieber V, Timmerman R, Tomé WA, Verellen D, Wang L, Yin FF (2010). Stereotactic body radiation therapy: the report of AAPM Task Group 101. Med Phys.

[23] Gardner EA, Sumanaweera TS, Blanck O, Iwamura AK, Steel JP, Dieterich S, Maguire P (2012). In vivo dose measurement using TLDs and MOSFET dosimeters for cardiac radiosurgery. J Appl Clin Med Phys.

[24] Ernst F, Bruder R, Schlaefer A, Schweikard A (2011). Forecasting pulsatory motion for non-invasive cardiac radiosurgery: an analysis of algorithms from respiratory motion prediction. Int J Comput Assist Radiol Surg.

[25] Kim DW, Cho LC, Straka C, Christie A, Lotan Y, Pistenmaa D, Kavanagh BD, Nanda A, Kueplian P, Brindle J, Cooley S, Perkins A, Raben D, Xie XJ, Timmerman RD (2014). Predictors of rectal tolerance observed in a dose-escalated phase 1-2 trial of stereotactic body radiation therapy for prostate cancer. Int J Radiat Oncol Biol Phys.

